# HCV and Oxidative Stress: Implications for HCV Life Cycle and HCV-Associated Pathogenesis

**DOI:** 10.1155/2016/9012580

**Published:** 2016-02-03

**Authors:** Regina Medvedev, Daniela Ploen, Eberhard Hildt

**Affiliations:** ^1^Department of Virology, Paul-Ehrlich-Institut, Paul-Ehrlich-Straße 51–59, 63225 Langen, Germany; ^2^Deutsches Zentrum für Infektionsforschung (DZIF), Gießen-Marburg-Langen, 63225 Langen, Germany

## Abstract

HCV (hepatitis C virus) is a member of the Flaviviridae family that contains a single-stranded positive-sense RNA genome of approximately 9600 bases. HCV is a major causative agent for chronic liver diseases such as steatosis, fibrosis, cirrhosis, and hepatocellular carcinoma which are caused by multifactorial processes. Elevated levels of reactive oxygen species (ROS) are considered as a major factor contributing to HCV-associated pathogenesis. This review summarizes the mechanisms involved in formation of ROS in HCV replicating cells and describes the interference of HCV with ROS detoxifying systems. The relevance of ROS for HCV-associated pathogenesis is reviewed with a focus on the interference of elevated ROS levels with processes controlling liver regeneration. The overview about the impact of ROS for the viral life cycle is focused on the relevance of autophagy for the HCV life cycle and the crosstalk between HCV, elevated ROS levels, and the induction of autophagy.

## 1. Introduction

At present, almost 2% of the world population suffers from chronic infection with HCV.HCV (hepatitis C virus) is a major cause of chronic hepatitis, steatosis, fibrosis, liver cirrhosis, and hepatocellular carcinoma. HCV belongs to the Flaviviridae family and contains a single-stranded positive-sense RNA genome of approximately 9600 bases [1, 2]. The viral genome is translated into a polyprotein that encompasses about 3100 amino acids. Cellular and viral proteases process the polyprotein cotranslationally and/or posttranslationally. After proteolytic processing of the amino terminal part of the polyprotein, the three structural proteins, core and the envelope proteins E1 and E2, are formed. The pore forming p7 protein (for a recent review, see [3]) stands between the structural proteins and the nonstructural proteins (NS), NS2, NS3, NS4A, NS4B, NS5A, and NS5B [4–6]. The nonstructural proteins form the replicon complex, localized on the cytoplasmic face of the ER. NS5B is the RNA-dependent RNA polymerase [7] and NS2 and NS3 represent viral proteases. NS3 in addition displays RNA helicase/NTPase activity; NS5A has RNA binding capacity, induces lipid droplet formation, and affects a variety of signal transduction cascades. HCV replication takes place at specialized rearranged intracellular membranes derived from the ER (endoplasmic reticulum), the so-called membranous web [5, 8]. Genome replication does not require the presence of the viral structural proteins enabling the establishment of subgenomic replicons that allow the analysis of genome replication in the absence of virion formation. Before cell culture systems based on the JFH-1 genome [9–11] and Huh7.5 cells were established, the subgenomic replicons were an extremely helpful system to study many aspects of the viral life cycle involved in genome replication [12].

The HCV life cycle is tightly associated with hepatocyte lipid metabolism [13]. The membranous web is enriched in proteins involved in very low density lipoproteins (VLDL) assembly, that is, apolipoprotein (apo)B, apoE, and microsomal triglyceride transfer protein (MTP) [14–17]. Lipid droplets (LDs), organelles for the intracellular storage of neutral lipids, serve as an assembly platform for HCV and thus play an important role for the morphogenesis of HCV [18–20]. LD formation can be induced by core and NS5A. In HCV replicating cells, core is addressed to LDs via diacylglycerol transferase 1 [21, 22]. HCV assembly and morphogenesis start on the surface of LDs.

There are many reports describing elevated ROS (reactive oxygen species) levels in HCV replicating cells and in liver tissue and lymphocytes derived from HCV-infected patients [23–27]. In this review, the term ROS encompasses various radicals such as the superoxide anion (O_2_^−•^), the hydroxyl radical (HO^•^), or hydrogen peroxide (H_2_O_2_).

In the liver, high amounts of the antioxidant glutathione (GSH) are found, which plays an important role in phase II metabolism of xenobiotics. In many patients suffering from chronic HCV infection, reduced levels of GSH are found in the serum and in the liver. Moreover, the ratio between the oxidized form (GSSG) and the reduced form (GSH) is elevated, reflecting the partially depleted antioxidant potential [28].

Eukaryotic cells have evolved a variety of strategies to detoxify ROS. Among these are reducing components such as glutathione or small oxidoreductases such as thioredoxin or enzymes that directly detoxify ROS, that is, peroxidases. A central role for the expression of a variety of cytoprotective enzymes plays the transcription factor Nrf2 (NF-E2-related factor 2) [29–31]. In its inactive state, Nrf2 is complexed with its endogenous inhibitor Keap1 (Kelch-like ECH-associated protein 1). This complex is rapidly degraded by the proteasomal system. Elevated levels of radicals or electrophiles affect intracellular SH-groups in Keap1, leading to their modification, which finally leads to a conformational change of Keap1 which results in the release of Nrf2 [32]. Nrf2 enters the nucleus and binds as a heterodimer with sMaf proteins to a conserved sequence in the promoter of cytoprotective genes, the antioxidant response element (ARE), and thereby induces the expression of genes harbouring AREs in their promoter. Nrf2-deficient mice have an impaired liver regeneration [33, 34]. The lack of Nrf2 leads to a diminished expression of cytoprotective genes and therefore to elevated ROS levels triggering an activation of JNK [35]. Activated JNK1/2 leads to a Ser/Thr phosphorylation of IRS1/2 which interrupts insulin receptor-dependent activation of proliferative pathways such as activation of protein kinase B (AKT/PKB). This would require tyrosine phosphorylation of IRS1/2 [36].

On the other hand, constitutive activation of Nrf2 also negatively affects liver regeneration. In transgenic mice, overexpressing caNrf2 liver regeneration is impaired due to impaired proliferation and enhanced apoptosis. An upregulation of the cyclin-dependent kinase inhibitor p15 and of the proapoptotic protein Bim can be observed. There is evidence for a crosstalk between Nrf2 and NF-*κ*B [37, 38]. In neuronal cells, it was observed that activation of Nrf2 is associated with a decreased activation of NF-*κ*B, leading to an elevated sensitivity for apoptotic stimuli [39].

This review aims to provide an overview about the modulation of radical generating pathways in HCV replicating cells and to summarize the interference of HCV with ROS inactivating mechanisms. The relevance of ROS for the HCV life cycle which is focused on the crosstalk of ROS with the induction and the impact of autophagy for the life cycle of HCV is discussed. Finally, the importance of ROS for the onset of HCV-associated pathogenesis with a focus on the interference with pathways affecting liver regeneration is summarized.

## 2. Sources of Reactive Oxygen Species (ROS) in HCV Replicating Cells

### 2.1. Increased Mitochondrial ROS Production

Mitochondria play a crucial role for the production of ROS in HCV-infected cells [26]. HCV core protein is considered as a major regulator affecting the release of ROS from mitochondria [40]. The full-length core protein (aa1–191) and the mature form of core (aa1–173) have been described to directly associate with the outer mitochondrial membrane [41–43]. The interaction with the outer mitochondrial membrane is mediated via the C-terminal hydrophobic motif [41, 44]. Moreover, core was found in the mitochondria-associated membrane (MAM) fraction: here a close contact between the membrane of the endoplasmic reticulum (ER) and the mitochondrion exists [41, 45]. Moreover, there is a report that described, based on electron microscopy analysis, an association of core with the inner mitochondrial membrane [46]. On the other hand, a detailed analysis based on confocal immunofluorescence microscopy of HCVcc-infected Huh7.5 cells found no evidence for a direct interaction of core with mitochondria [47, 48], while others described that, regardless of whether core was selectively overexpressed or produced in the context of HCV replication in JFH-1 or J6 positive cells, core is directly associated with the mitochondrion [49]. The detailed analysis of the association of core with the mitochondria is hampered by the fact that much higher amounts of core are localized at the ER and on the surface of LDs. This makes it difficult to discriminate whether a real mitochondrial association is observed or the observation reflects the much stronger signal based on the MAM localization of core.

In addition to core, a fraction of NS3/NS4A, a viral protease complex, is associated with the outer mitochondrial membrane where it cleaves MAVS (mitochondrial antiviral signaling protein). MAVS is tail-anchored at the outer mitochondrial membrane [50, 51]. MAVS [52] is an essential component of the innate immune response pathway: dsRNA is recognized by RIG-I which leads via activation of IRF3 to the expression of IFN-beta.

### 2.2. Relevance of Elevated Cytosolic Ca^2+^ Levels for Induction of ROS Formation

HCV replication and selective core overexpression induce ER stress [53], leading to Ca^2+^ release from the ER into the cytoplasm [54–57]. In parallel, core inhibits the sarcoplasmic/endoplasmic reticulum calcium ATPase (SERCA) and thereby further contributes to an elevated cytoplasmic Ca^2+^ level. NS5A acts as an additional factor perturbing Ca^2+^ homeostasis by triggering the release of Ca^2+^ from the ER in the cytoplasm [57].

The VDAC (voltage-dependent anion channel) is the major component of the mitochondrial permeability transition (MPT) pore. Interacting with the VDAC core sensitizes the MPT pore and increases the mitochondrial Ca^2+^ uniporter activity [59], leading to increased Ca^2+^ influx in the mitochondria. In vitro experiments support this. Addition of core to purified mitochondria is sufficient to increase Ca^2+^ uptake and to induce ROS production [44, 59, 60].

This reflects a* vicious circle*: the mitochondrial sensitization to Ca^2+^ along with the opening of the MPT pore triggered by core leads to increased ROS levels which in turn induce MPT pore opening.

### 2.3. Mechanism of Ca^2+^ Mediated Induction of ROS Formation

The increased uptake of Ca^2+^ into mitochondria leads to perturbation of the electron transport chain [44, 54] by inhibition of the electron transport complex I (NADH:ubiquinone oxidoreductase). Electrons are likely to leak from the electron transport chain (ETC) favouring O_2_^•−^/H_2_O_2_ formation. So [61, 62], the elevated ROS production is based on complex 1 substrates due to decreased complex I activity but not due to a direct interaction of the electron transport complexes with core or other HCV proteins. While the activity/functionality of the complexes II and III are not affected by HCV, the complex IV (cytochrome c oxidase) is the other complex affected by HCV and so it contributes to increased ROS formation by the mitochondria.

Pharmacological interference with ER/mitochondrial Ca^2+^ flux normalizes the electron transport chain complex I activity, restores mitochondrial membrane potential, and normalizes ROS production [54]. Comparable effects can be achieved by chelators [60], indicating the prominent role of Ca^2+^ for mitochondrial dysfunction.

Apart from the direct effects on the respiratory chain, enzymes of the TCA and of the lipid beta oxidation are affected in their functionality [63, 64]. By the resulting decreased amount of NADH/FADH equivalents, the electron transport chain and subsequently the mitochondrial membrane potential are affected, contributing to the increased ROS formation.

Decreased levels of prohibitin are described to lead to impaired assembly and functionality of the complexes of the respiratory chain [65]. The elevated level of prohibitin in HCV replicating cells could reflect the disturbed function of the complexes I and IV of the electron transport chain [66, 67].

## 3. Impact of NADPH Oxidases for ROS Formation in HCV Replicating Cells

NADPH oxidases (NOX) are multimeric transmembrane enzyme complexes that generate superoxide anions (O_2_^•−^) and hydrogen peroxide (H_2_O_2_) from molecular oxygen, using NADPH as electron donor (recent review: [68]). In mammals, the NOX family encompasses 7 members. In the liver NOX1, NOX2, and NOX4 are found. In case of NOX2, a heterodimeric transmembrane protein encompassing the regulatory subunit p22phox and the catalytic subunit gp91phox, Rac is a further component of the active enzyme complex. Further regulatory components in addition to Rac are p47phox, p40phox, and p67phox. Upon stimulation, these regulatory factors translocate from the cytoplasm to the membrane-bound heterodimeric enzyme complex. In contrast to NOX2, NOX4 requires only p22phox for its enzymatic activity [69, 70].

In the liver, NOX is functionally expressed both in the phagocytic form and in the nonphagocytic form. Various NOX isoforms such as NOX1, NOX2, and NOX4 are distinctively expressed in the specific cell types, including Kupffer cells (KCs), HSCs, endothelial cells (ECs), hepatocytes, and infiltrating leukocytes in the liver [68]. NOX2-derived ROS act in the immune defense in neutrophils and phagocytes.

NOX4 is ubiquitously expressed and inducible. In HCV negative cells NOX4 is found in the cytoplasm and in the nucleus; in HCV replicating cells NOX4 expression is increased and is found preferentially in the nucleus and at the ER [71]. Nuclear formation of ROS by the nuclear NOX4 favours DNA damage.

HCV induces the NOX4 expression: core induces TGF-beta which triggers expression of NOX4. Elevated levels of NOX1 are found in Huh7.5 cells as well as in liver samples from HCV positive patients [72].

## 4. ER Stress, UPR, and Further Sources of ROS in HCV Replicating Cells

The unfolded protein response (UPR) leads to increased expression of PDI and ER oxidoreductins (Ero1). Ero1 is involved in disulfide bond formation leading to H_2_O_2_ production as by-product [73]. The induction of ER stress by HCV replication [74, 75] or selective overexpression of HCV structural proteins, E1 and E2 [76], or nonstructural protein, NS4B [77] (overview in [78]), is described. Moreover, Ero1 is involved in the control of Ca^2+^ release from mitochondria [79].

For core-dependent induction of ER stress it was described that both the EIF2AK3 and ATF6 pathways of the unfolded protein response (UPR) were activated by HCV core protein. This contributes to HCV-dependent induction of autophagy [80]. Moreover, HCV replication is increased by upregulation of both WT-PGC-1*α* and L-PGC-1*α* through an ER stress-mediated, phosphorylated CREB-dependent pathway [75].

The expression of the ER residing cytochrome P450 2E1 (CYP2E1) is increased in HCV replicating cells and in HCV patients [81]. CYP2E1 is part of the microsomal ethanol oxidizing system (MEOS) which metabolizes ethanol to acetaldehyde in the presence of oxygen and NADPH. CYP2E1 has a significant NADPH oxidase activity, leading to the generation of large quantities of O_2_^•−^, H_2_O_2_, and hydroxyethyl radicals (recent review: [82]). Whether ROS triggers the relocalization of CYP2E1 to the mitochondrion is discussed [83].

## 5. Interference of HCV with the Nrf2/ARE Pathway

The expression of a variety of cytoprotective genes is controlled by the transcription factor Nrf2. Upon activation Nrf2 is released from the complex with Keap1 and enters the nucleus where it binds as a heterodimer with sMaf proteins to the antioxidant response element, a short cis-acting element which is found in the promoter of Nrf2-dependent genes and induces the expression of ARE-dependent genes [29, 32]. ARE-dependent genes are involved in the removal of ROI, that is, glutathione-dependent peroxidase (GPx), in the glutathione metabolism, in detoxification of electrophiles by generation of reductive equivalents, or in the removal of misfolded proteins. Examples for ARE-regulated genes are NAD(P)H:quinone oxidoreductase 1 (NQO1), glutathione peroxidase (pHGPx), the regulatory and catalytic subunits of glutamate-cysteine ligase (GCLM and GCLC) or glutathione S-transferases (GST), or catalytic units of the constitutive proteasome as PSMB5 [84].

There are conflicting results about the interference of HCV with the Nrf2/ARE system. In a study published by Burdette et al. [85], based on HCV (JFH-1)-infected Huh7 cells, an induction of Nrf2 expression and activation of Nrf2 were described. PDTC as radical scavenger and Ca^2+^ chelating agents abolished HCV-dependent activation of Nrf2. Based on a screen of various kinase inhibitors, p38MAPK and Janus kinase were identified to mediate Nrf2 phosphorylation/activation in HCV replicating Huh7 cells.

In a recent study [86], an increased expression of Nrf2 and nuclear localization of Nrf2 were described for HCV (JFH-1)-infected Huh7.5.1 cells. The HCV-dependent induction of Nrf2 expression is associated with an inhibitory phosphorylation of GSK3-beta which is necessary and sufficient for the induction of Nrf2 by HCV. Moreover, a direct association between GSK3-beta and Nrf2 was described in this study.

Cotransfection of Huh7 cells with expression constructs encoding HCV core, E1, E2, NS4B, or NS5A and an ARE-dependent luciferase reporter construct indicates that selective overproduction of these proteins is able to trigger activation of Nrf2. Further analyses revealed that the activation of Nrf2 by these proteins is due to elevated ROS levels and is transduced by PKC [87].

In contrast to these observations, Carvajal-Yepes et al. [58] found in HCV (JFH-1 and JFH-1/J6) replicating cells and in HCV-infected primary human hepatocytes an inhibitory effect on the activation of Nrf2 and the induction of Nrf2/ARE-dependent genes. In HCV replicating cells, a translocation of the sMafs from the nucleus to the replicon complex occurs, where the sMafs bind to NS3 and thereby are withdrawn from the nucleus. This process depends on the presence of core. In core-deficient mutants this effect is not observed. The replicon-bound sMaf proteins bind Nrf2 and thereby prevent its translocation into the nucleus, leading to an inhibition of Nrf2. In accordance with this, increased sensitivity of HCV replicating cells to ROS-dependent modifications of proteins and DNA (8-OHdG formation) was observed in this study.

These data are in accordance with a transcriptome analysis of HCV replicating cells which revealed a significant reduction of the expression of a variety of Nrf2-dependent genes such as NQO1, epoxide hydrolase 1 (ephx1), catalase (cat), and glutamate-cysteine ligase catalytic subunit (GCLC) and other enzymes of the glutathione metabolism [88]. Moreover, in liver biopsies derived from HCV-infected patients, a decreased expression of heme oxygenase 1 (HO-1), which is Nrf2-dependent regulated, was observed [89, 90]. In contrast to this, in HH4 hepatocytes expressing HCV genome genotype 1a, an increased level of intracellular glutathione and elevated expression of glutathione-S-transferase 3 (GST3) and of metallothionein were described [91]. A recent report described an HCV-dependent induction of glutathione peroxidase 4 (GPx4) in HCV replicating cells and in liver biopsies. The induction is triggered by NS5A via phosphatidylinositol-3-kinase [92].

## 6. Relevance of ROS for the HCV Life Cycle

The relevance of HCV-dependent induction of oxidative stress with respect to viral genome replication is controversially discussed. On one hand, there are reports describing an inhibitory effect of elevated ROS levels on HCV replication [93, 94] and on the other hand there are reports describing Pycnogenol, a pine extract, which has antioxidant effects and leads to reduced ROS levels and impaired HCV replication [95].

The impact of autophagy on the HCV life cycle is established (for a recent overview, see [96]). This review here focuses on the crosstalk between HCV-dependent induction of ROS, autophagy, and virus morphogenesis.

## 7. Autophagy

Autophagy, also considered as a “self-eating” process, is a highly conserved and regulated degradation mechanism to maintain cellular homeostasis. Through engulfment of damaged cytoplasmic organelles and protein aggregates, autophagy plays an essential role as a cellular stress response to counteract, for example, ER stress, nutrient deprivation, or a pathogen infection to ensure cell survival [97–99]. Autophagosome formation can be divided into three major steps: the initiation, which begins with the formation of the phagophore assembly site or isolation membranes, the nucleation, and the expansion and enclosure to form double lipid bilayer membrane-bound autophagosomes with an average size of 300–900 nm [100]. Mature autophagosomes then fuse with lysosomes to form autophagolysosomes, where the sequestered cargo is subsequently degraded [101].

The serine/threonine kinase mammalian target of rapamycin (mTOR), a central regulator of the nutrient-sensing pathway, maintains the balance between degradation and synthesis, thereby controlling the growth and starvation response depending on the energy level of the cell [102]. Under nutrient-rich conditions, mTOR suppresses autophagy by phosphorylation of Unc-like kinase 1 und 2 (ULK1/2 complex) [103, 104]. Upon starvation, AMP-activated protein kinase inhibits the mTOR kinase activity, causing an activation of autophagy [105]. This canonical process is regulated by more than 30 autophagy-related genes (Atg) [106]. Four functional units of Atg proteins are involved in the regulation: the Unc-51-like kinase complex (ULK1/2-Atg13-FIP200-ATG101), the class III phosphatidylinositol-3-OH kinase complex (PI3K-Vps-15-Beclin-1-Atg14), and the two ubiquitin-like conjugation systems Atg12 (Atg5-Atg12-Atg16L) and Atg8/LC3 (Atg4-Atg3-LC3) [107]. Upon autophagy induction, the ULK1/2 complex translocates from the cytoplasm to special ER domains, forming preautophagosomal structure- (PAS-) like structures [108]. Then, in the nucleation step, the activated ULK complex recruits the class III PI3K complex to catalyze the production of autophagosome-specific phosphatidylinositol-3-phosphate (PI3P) [109]. The PI3P effectors DFCP1 (double-FYVE-containing protein 1) and WIPI (WD-repeat protein interacting with phosphoinositides) are recruited, leading to a formation of an ER-associated Ω-like shape, the omegasome, to create the isolation membrane (IM) [110]. Finally, the isolation membrane expands to form the enclosed autophagosome. This process requires the two ubiquitin-like conjugation systems Atg5-Atg12-Atg16L and Atg4-Atg3-LC3. First, Atg12 forms a conjugate with Atg5 by activation through Atg7 (E1-like enzyme) and Atg10 (E2-like enzyme). The Atg12-Atg5 conjugate then interacts with Atg16L to form the Atg5-Atg12-Atg16L complex. This complex serves as an E3-ligase to facilitate the conjugation of cytosolic microtubule-associated protein 1 light chain 3 (LC3) to phosphatidylethanolamine, producing the membrane-bound, lipidated form LC3-II [111]. Finally, the autophagosomes can either fuse with endosomes, forming the hybrid organelle, the amphisome, before fusing with the lysosome or directly fuse with lysosomes, forming the autophagolysosomes to degrade and recycle the sequestered materials [99]. Despite the assumption that the ER is the main membrane source for autophagosome formation, membranes can further originate from ER mitochondria junctions [112], mitochondria [113], Golgi compartment [114], endosomes, and plasma membrane [115].

For the autophagosome-lysosome fusion, the autophagosomal SNARE (soluble N-ethylmaleimide-sensitive factor attachment) protein syntaxin 17 (Stx17) plays a central role [116]. Therefore, Itakura et al. described that Stx17 only translocates on the outer membrane of completed autophagosomes, but not on phagophores, to avoid early fusion with the lysosome. Another regulator of the autophagolysosome formation is the PLEKHM1, (pleckstrin homology domain containing protein family member 1) which controls the fusion through binding to LC3 and HOPS (homotypic fusion and protein sorting) complex, as genetic loss of PLEKHM1 leads to an impaired autophagosome-lysosome fusion [117]. Furthermore, the small GTPase Rab7 is involved in the fusion of the autophagosome with the lysosome [118].

Autophagy is a converging point of different stimuli [119]. As already mentioned, autophagy is a very sensitive process underlying cell responses induced by nearly any stressful condition which influences the cellular homeostasis [106]. Thereby, cells coordinate energy and maintain the nutrient pool for metabolic reactions. If the cell is not able to perpetuate the rate of protein synthesis or to provide the required amount of ATP, autophagy is induced [119]. Furthermore, ROS have been indicated as early inducers of autophagy upon nutrient starvation [120, 121]. It is still ambiguous which reactive species promotes the process, as several publications either indicate H_2_O_2_ as the molecule generated directly after nutrient deprivation [122, 123] or propose O_2_^•−^ as the primary ROS inducing autophagy [124], whereas others just suppose that ROS are indispensable for autophagy, given that treatment with antioxidants partly or completely reverses the mechanism [125].

Although more insights into the complex organization and regulation of autophagy have been gained, further analysis will be needed to better understand the membrane rearrangement processes.

## 8. Crosstalk Autophagy and the Keap1-Nrf2 Pathway

Autophagy is a major catabolic process that degrades cytoplasmic constituents for the clearance of long-lived or misfolded proteins and damaged organelles. This process functions to keep the cellular homeostasis and to protect the cell against oxidative stress. Starvation as well as oxidative and ER stress can induce autophagy [119, 126]. Another cellular protection system against oxidative and electrophile stress is the Keap1-Nrf2-ARE pathway [127–130].

The crosstalk between autophagy and the Keap1-Nrf2-ARE pathway was uncovered in 2010, when several groups verified the direct interaction between p62 and Keap1 [131–136]. p62 is a stress-inducible cellular protein that has various domains that mediate its interactions with several binding partners, such as LC3-interacting domain (LIR), Keap1-interacting domain (KIR), and ubiquitin-associated (UBA) domain. Furthermore, it operates as a signaling hub and regulates divers' stress responses [137].

For selective autophagy, p62 plays an important role as autophagy adaptor and binds to ubiquitylated cargo (UBA domain) and guides the cargo towards autophagosomal degradation by interacting with LC3 (LIR domain) [138].

Furthermore, p62 interacts with Keap1 (KIR domain), linking the autophagic- and Keap1-Nrf2-pathway [136]. Thereby, the phosphorylation of p62 of Serine 351 of the KIR increases p62's binding affinity for Keap1 and for this reason competitively inhibits the Keap1-Nrf2 interaction. Hence, Keap1 is sequestered into the autophagosome, leading to an impaired ubiquitylation and subsequent activation of Nrf2 [139]. The kinase phosphorylating p62 on S351 is still unknown. Due to controversial observations, the detailed Keap1-p62 interaction mechanism remains to be solved [133, 134, 139]. Furthermore, it was observed that p62 regulates the degradation of Keap1 by controlling the Keap1 turnover [135].

**Figure 1:**
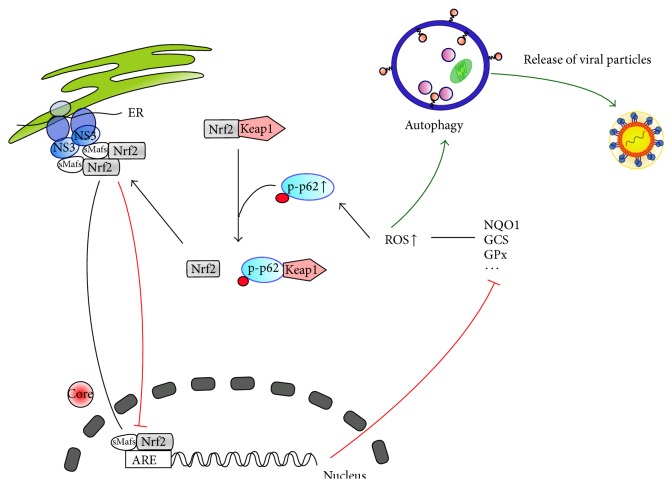
Hypothetical model summarizing the interplay between Nrf2, phosphor-p62, ROS, and autophagy. As described by Carvajal-Yepes et al. [58], Nrf2/ARE-signaling in HCV positive cells is impaired by translocation of sMaf from the nucleus to NS3 in the replicon complexes on the cytoplasmic face of the ER. This leads to the sequestration of Nrf2 to the replicon complex-bound sMaf and prevents the phospho-p62-dependent released Nrf2 from the entry in the nucleus and thereby inhibits the induction of Nrf2/ARE-dependent genes. This would contribute to an impaired elimination of ROS that contribute to the induction of autophagy that is crucial for the HCV life cycle.

One can speculate whether the complex mechanism leading to impaired Nrf2/ARE-signaling in HCV positive cells by translocation of sMaf from the nucleus by NS3 to the replicon complexes on the cytoplasmic face of the ER and subsequent sequestration of Nrf2 to the replicon complex-bound sMaf prevents the phospho-p62-dependent released Nrf2 from the entry in the nucleus and thereby inhibits the induction of Nrf2/ARE-dependent genes. This could contribute to an impaired elimination of ROS that contribute to the induction of autophagy which is crucial for the HCV life cycle. This hypothesis is graphically summarized in Figure 1 [58].

It is known that elevated ROS levels can induce the autophagic machinery [120]. In line with this, several studies have shown that in HCV replicating cells the unfolded protein response (UPR) and the autophagic pathway are activated, although the data are conflicting and the detailed mechanism of activation remains to be solved [96].

Several pathways are affected upon HCV infection to result in an induction of autophagy. As already mentioned, HCV infection is associated with oxidative stress and elevated ROS levels. Thereupon, the direct PERK-Nrf2 or the indirect IRE1*α*-JNK-Nrf2 pathway causes the expression of detoxifying genes [140]. Furthermore, it is supposed that HCV induces ER stress upon activation of UPR in vivo [141] and in vitro [142–145]. Several reports have shown that NS4B plays a central role in UPR and autophagy induction. Li et al. identified that NS4B can induce UPR through activation of the IRE1 or ATF6 pathway, as it interferes with Ca^2+^-homeostasis, leading to elevated ROS levels [146]. Additionally, Su et al. reported that NS4B interacts with Rab5 and Vps34 and thereby triggers autophagy induction [147]. Furthermore, NS5A was also described to induce autophagy [148]. Moreover, Grégoire et al. demonstrated that different RNA viruses, including HCV, can modulate autophagy by interacting with the human immunity-associated GTPase family M (IRGM) [149]. Taken together, these results showed that a variety of signaling pathways are involved in HCV-induced autophagy. Nevertheless, further investigations are required to understand the complex interplay between HCV and the cellular stress response.

Several studies depicted that autophagy plays a crucial role for the viral life cycle, that is, for the membranous web formation and translation of incoming RNA, replication, and virus release [80, 150, 151]; for recent reviews, see [96, 152, 153].

HCV replication takes place in replication complexes at the so-called membranous web, which consists of ER-derived single-, double-, and multimembrane vesicles and lipid droplets [154–156]. Therefore, an interaction between NS proteins and the autophagic machinery is essential for the reconstruction of the intracellular membranes for the formation of the membranous web [147, 154, 155, 157–165].

The data regarding the role of autophagy for HCV replication are contradictory and yet not fully understood. Several groups demonstrated that HCV induces complete autophagy via the UPR to promote viral replication [142, 143, 145, 155, 166–168], whereas Sir et al. showed contradictory results, reporting that autophagosome maturation seems to be incomplete in HCV replicating cells [165]. A possible explanation for the discrepancy could be the use of different cell culture systems for the studies.

The assembly and release of viral particles are closely linked to the VLDL synthesis and occur via the secretory pathway [169]. Nevertheless, the exact mechanism still remains unclear. Involvement of the autophagic pathway in the release of lipoviral particles was described [16, 17]. Furthermore, Tanida et al. reported that silencing of Atg7 and Beclin-1 inhibits the release of infectious viral particles but does not influence the amount of intracellular viral RNA [167]. Additionally, Grégoire et al. revealed that IRGM interacts with Atg5, Atg10, LC3, and SH3GBL1 and participates in the induction of autophagy and release of viral particles [149, 170].

Summing up, autophagy interferes with various steps of the viral life cycle to promote a permanent viral infection. In light of this, a complex interplay between HCV-induced oxidative stress and the Nrf2/ARE-signaling preserves elevated ROS levels which are necessary for induction of autophagy for HCV life cycle.

## 9. HCV-Induced Insulin Resistance

HCV infection often comes along with a variety of liver diseases. Oxidative stress is known to play a central role in many of them, as HCV-induced oxidative stress leads to liver damage. A clear correlation between oxidative stress and insulin resistance was reported [171]. Nrf2 is known to be an important regulator of the cellular redox homeostasis, inducing the expression of cytoprotective genes upon elevated ROS levels [34]. Additionally, Beyer et al. identified a further role of Nrf2 in tissue repair [33]. Liver regeneration in Nrf2 knockout mice is delayed after partial hepatectomy or CCl_4_-dependent induction of liver damage. This effect was caused by an oxidative stress-mediated resistance to insulin and insulin-like growth factor. Previous investigations have shown an inhibitory effect of ROS on the insulin/insulin growth factor-1 (IGF-1) receptor signaling. This effect was mediated by activation of serine/threonine kinases, that is, JNK, by ROS, which phosphorylate IRS-1 and IRS-2 [172, 173]. Ser/Thr phosphorylation of IRS-1 and IRS-2 leads to dissociation from the insulin receptor, leading to a reduced tyrosine phosphorylation of IRS-1and IRS-2 by the receptor kinase. This results in reduced activation of downstream targets, that is, AKT and p38 [174, 175]. As Nrf2 signaling is impaired upon HCV infection, resulting in elevated ROS levels, this leads to ROS-dependent activation of JNK. Activated JNK phosphorylates IRS1/2 on Ser/Thr residues and thereby impairs insulin signaling.

Shlomai et al. observed an increased expression of peroxisome proliferator-activated receptor-gamma coactivator 1*α* (PGC-1*α*), which could also contribute to HCV-induced insulin resistance [176]. PGC-1*α* is involved in the induction of insulin resistance upon oxidative stress and is a transcriptional cofactor activating the expression of genes involved in the initiation of gluconeogenesis, that is, upregulation of glucose-6-phosphatase (G6Pase), resulting in elevated glucose production [177–179]. Furthermore, an enhanced fatty acid uptake or upregulation of genes involved in lipid and cholesterol synthesis may contribute to oxidative stress-induced insulin resistance [180, 181].

## 10. Outlook

At present, there exists a lot of information about the fact that HCV infection increases oxidative stress. But the underlying mechanisms and the balance between ROS generating and ROS detoxifying pathways and the regulation of this balance by HCV are not completely understood as reflected by a lot of seemingly contradictory results. To clarify the interplay between these pathways and to reveal the relevance of host factors and of viral factors regarding differences between the HCV genotypes for ROS production/detoxification will be a challenge for the next years. Based on this, whether modulation of this interplay has an impact on the design of antiviral therapy will be analyzed.
